# Prediction of outcome in adults with severe falciparum malaria: a new scoring system

**DOI:** 10.1186/1475-2875-6-24

**Published:** 2007-02-27

**Authors:** Saroj K Mishra, Pinaki Panigrahi, Rajalaxmi Mishra, Sanjib Mohanty

**Affiliations:** 1 Department of Internal Medicine, Ispat General Hospital, F-139, Sector-19, Rourkela, 769 005, Orissa, India; 2 Department of Pediatrics, Epidemiology and Preventive Medicine, University of Maryland School of Medicine, Baltimore, MD, USA; 3 Department of Mathematics, Sushilavati Government Women's College, Rourkela, Orissa, India

## Abstract

**Background:**

Mortality of falciparum malaria is related to the presence of severe complications. However, no scoring system is available to predict outcome of these patients. The aim of this paper was to devise a simple and reliable malaria prognosis score (MPS) to predict the outcome of adults with severe malaria.

**Methods:**

All slide-positive severe falciparum malaria patients admitted to Ispat General Hospital were studied. Eight clinical parameters that may potentially differentiate or influence the outcome were identified to predict recovery or death

**Results:**

Of 248 severe malaria cases, 35 died. There were 212 adults (34 deaths) and 36 children (one death). The malaria score for adults was (MSA) = 1(severe anaemia) + 2 (acute renal failure) + 3(Respiratory distress) +4 (cerebral malaria). The MSA ranges from 0 to 10. The mortality was 2% for MSA 0 – 2; 10% for MSA 3–4, 40% for MSA 5–6 and 90% for MSA 7 or more. The sensitivity is 89.9% and positive predictive value is 94.1% when 5 is taken as the cut off value.

**Conclusion:**

MSA is a simple and sensitive predictor. It can be administered rapidly and repeatedly to prognosticate the outcome of severe malaria in adults. It can help the treating doctor to assess the patient as well as to communicate to the relatives of the patients about prognosis. The score needs revalidation in other geographical areas.

## Background

Falciparum malaria is a leading cause of mortality and morbidity in developing countries [1]. Severe falciparum malariais associated with various complications. The WHO enumerates a list of complications [1], but the importance of each complication has not been assigned. It is well known that the presence of multiple complications results in increased mortality. The presence of pregnancy makes the outcome disastrous [2]. Similarly, Marsh et al reported respiratory distress as an indicator of life-threatening malaria in African children [3,4]. Molyneux et al described the clinical and prognostic indicators of cerebral malaria in children [5]. However, no prediction scoring system is yet available to prognosticate the outcome in severe malaria among adult patients.

A simple scoring system is described to predict mortality in any severe and complicated malaria, based on available variables at the bedside with minimum laboratory parameters. The Malaria Score for adults (MSA) can be utilized at any time, and not as a "once only" predictor.

## Methods

### Study area

The study was carried out at Ispat General Hospital, Rourkela, in the district of Sundergarh, in the northwestern part of State of Orissa, India. It is a 700 bed hospital, which caters to the medical needs of more than 500,000 populations. Being a large and well-equipped hospital, over the years it has established as a tertiary care center for seriously ill patients from the adjoining districts of Orissa, Jharkhand, Chhatisgarh, Madhya Pradesh and Bihar.

### Patients

This hospital-based study was conducted on patients over 12 years of age presenting with falciparum malaria who met the WHO criteria of severe and complicated falciparum malaria [1]. It includes cerebral malaria (unrousable coma), severe anaemia (haemoglobin < 5 g/dL), renal failure (serum creatinine > 3 mg/dl), adult respiratory distress syndrome, hypoglycaemia (glucose < 40 mg/dl), circulatory collapse or shock, disseminated intravascular coagulation, repeated generalized convulsions, acidosis (pH < 7.25), macroscopic haemoglobinuria, hyperparasitemia (> 5%) or jaundice (bilirubin > 3 mg/dL). All the patients were symptomatic.

A detailed clinical evaluation was made with particular attention to neurological status, respiratory, renal condition and other complications. A sample of 5 ml heparinized venous blood was collected for determination of plasma glucose, urea, serum creatinine, bicarbonate, serum electrolytes, bilirubin, alanine aminotransferase, hemoglobin, and for a total and differential leukocyte count and a parasite count. Soon after confirmation of the presence of asexual forms of *Plasmodium falciparum *in the peripheral blood smear, injection quinine dihydrochloride 10 mg/Kg body weight in 5% dextrose or in dextrose saline was infused over a period of 4 hours, every 8 hours, until the patient was able to take the drug orally. Oral quinine sulphate was given to complete the total duration of seven days therapy. Supportive measures like maintenance of hydration, antibiotics for any concurrent infections, blood transfusion, dialysis, ventilator support etc. were given according to individual needs. The patients who recovered and those who expired were grouped and the statistical analyses were performed [6].

### Statistical analysis

The clinical parameters associated with survival and deaths were compared by using) Chi-square (χ^2^) test with Fisher exact test where applicable; Odds ratio (OR) with 95% Confidence Interval. Those parameters which were significant were used for the multivariate analysis to get a linear discriminant equation showing the relationship between malaria score and the variables, i.e., a formula in the form of Malaria Prediction Score (MPS) = a + b_1_x_1 _+ b_2_x_2 _+ b_3_x_3 _+ ... + b_n_x_n_

where **a **= constant, **b**_**i **_= discriminant coefficients (i =,2,3............n) and **x**_**i **_= independent variables (i = 1,2,3 ... ... n).

## Results

The study to develop the score included 248 severe malaria patients with 35 deaths. There were 36 children (one death) and 212 adults (34 deaths). All the variables were analysed (viz., age, gender, presence of cerebral malaria, seizures, hyperpyrexia, hypotension, severeanaemia, haemolysis, acute renal failure, jaundice, hypoglycaemia, respiratory complications needing ventilator support and pregnancy. Of these, cerebral malaria, severe anaemia, acute renal failure, and need of ventilator were identified as indicators of high risk. The adults were more vulnerable than the children. Presence of cerebral malaria, severe anaemia, renal failure or respiratory distress needing ventilatory support were highly significant whereas adult age or pregnancy were significant to a lesser degree. There was no difference in the mortality between males and females. The presence of haemolysis, hypoglycaemia or hypotension were not significantly different in the two groups. After multiple regression analysis, the results were studied to identify as few variables as possible to predict the outcome:

The formula for Malaria prediction Score (MPS) thus evolved was as follows:

MPS = 2.13 + 0.02 × (Age) + 0.25 × (Creat) - 0.24 × (Hb)

+ 3.05 (Cerebral) + 0.8 (Pregnancy)+ 0.8(Ventilator)

(where, Age = age in years; Creatinine is in mg/dl, Haemoglobin in G/dl; Presence of Pregnancy, cerebral malaria or ventilatory support, when present = 1, when absent = 0.)

However, there were some practical problems in MPS : a) only one death from 36 children and b) the laboratory data for serum creatinine, bilirubin, haemoglobin and blood sugar must be available to get the MPS. This would delay in calculating the MPS, and thus it cannot be administered at the time of evaluation. Hence, the data from those patients aged above 12 years were analysed to develop the Malaria score to predict the outcome among adult patients with severe malaria. The clinical parameters of those who survived vs those who expired are given in Table 1. From the table it signifies the importance of four variables. Viz., cerebral malaria, acute renal failure, severe anaemia and repiratory failure requiring ventilator. The presence of hypoglycemia, jaundice, seizures or pregnancy did not adversely influence the survival.

**Table 1:** Comparison of different parameters among those who survived & those who died.

Parameters*	Expired (n = 34)	Recovered (n = 178)	OR (95% CI)	P** <
Cerebral malaria	32	89	16.0 (3.85–140)	0.000
Seizures	02	15	0.68 (0.07–3.16)	0.465^a^
Severe anaemia	12	27	3.05 (1.25–7.4)	0.006
Jaundice	12	88	0.56 (0.24–1.27)	0.131
Acute renal failure	13	22	4.39 (1.78–10.8)	0.001
Hypoglycaemia	01	04	1.32 (0.03–13.86)	0.807^a^
Ventilatory support	08	05	10.56 (2.77–43.88)	0.000^a^
Pregnancy	06	19	1.79 (0.54–5.20)	0.249^a^

* Described in the text **χ^2 ^test a: by Fisher exact test

So the score was accordingly modified (a) to include only the adults, and (b) the presence or absence of a clinical parameter was denoted as 1 or 0. Thus the malaria score for adults was (MSA) = 1(severe anaemia) + 2 (acute renal failure) + 3(Respiratory distress) + 4 (cerebral malaria). The MSA ranges from 0 to 10. The mortality was 2% for MSA 0 – 2; 10% for MSA 3–4, 40% for MSA 5–6 and 90% for MSA 7 or more. The sensitivity is 89.9%, specificity 70.6%, and positive predictive value is 94.1% when 5 is taken as the cut off value.

## Discussion

The clinical course of severe malaria is variable depending on the presence of one or several complications. The fatality rate of cerebral malaria, renal complications, acute lung injury is very high and they cannot be grouped together as similar to hypoglycaemia, hypotension, hyperpyrexia or seizure. It iscrucial to prognosticate the cases accordingly. As it was observed, the presence of haemolysis, hyperpyrexia, hypoglycaemia or hypotension alone was not a major factor; and not a single patient died due to any one of these. Moreover, combination if these also did not end with fatality. The prognostic indicators as suggested by Molyneux et al [5] for childhood cerebral malaria includes four neurological parameters (witnessed seizures, coma score of 0, signs of decerebration, absent corneal reflexes); and three laboratory parameters (blood glucose level below 2.2 mmol/l, parasitaemia > 10^6 ^ring forms, total leukocyte count > 15 × 10^9^/l). This index is applicable only for the cerebral malaria cases, and there is no indicator for other forms of severe malaria. Moreover, each of the parameter was given a uniform weightage of 1. The positive predictive value of 4 or more of the above for an unsatisfactory outcome was 83% but sensitivity was only 66%.

Lewallen et al described the retinal findings to predict the outcome in cerebral malaria [7]. Papilloedema and extramacular retinal oedema are prognostically significant. The relative risk of death in patients with papilloedema was 6.7 times more than the patients without.

Marsh et al stressed the importance of respiratory complications as a simple bedside prognostic indicator in children with cerebral malaria [3]. This could identify 84.4% fatal cases. In an editorial in the same issue it was commented that one cannot exclude the possibility of other respiratory illnesses and incidental *P. falciparum *parasitaemia. The definition of different degrees of severity of respiratory distress needs refinement. Many of the components of the clinical picture are subjective thus increasing the risk of inter observer variation [4]. In the present study, respiratory distress needing ventilatory support is considered which thus eliminated the observer bias. In most of the studies the attention is concentrated on cerebral malaria. Here all complications were analyzed and their contributing factors were taken into account. All the parameters are unambiguous and free from subjective error.

Presence of multiple complications made the management difficult. Even in the tertiary care centers the management becomes challenging as a number of complications can appear at the time of admission or even during the course of hospitalization. To predict the survival of the index case is therefore very difficult and demanding from the relatives of the patient. A guarded opinion is invariably put forth, depending on the personal experience, intuition, clinical acumen of the physician; as no predictive scoring system yet available. The literature is scant about the relative risk of the complications in malaria In this situation, the severity classification APACHE-II [8] may be used, but it is too exhaustive and cumbersome. So the need was felt to develop malaria prediction score for adults, which can be used even in a small town hospital, to decide the continuation of treatment or referral to a higher center.

In some clinical situations, scoring methods are available for prediction of fatality (viz. burns [9], acute pancreatitis [10], stroke [11] etc); where the aim is prediction of mortality and hospital stay. In the case of malaria, if one can predict the survival, it is usually a full cure with rare sequel. So it is very important to predict the case survival. A scoring system though statistically significant has ultimately limited clinical use if (i) it is too cumbersome and difficult to remember, (ii) it has too many subjective variables or (iii) it requires sophisticated variables. Thus, a simple scoring system is always sought, so that only simple calculations would be needed, making it readily applicable at the bedside. The laboratory tests are routine and readily available; the clinical parameters are unambiguous without any subjective variation. The sensitivity of MSA is 89.9% and positive predictive value is 94.1%. It predicts better than the Malawian prognostic index (for cerebral malaria) 83% sensitivity of 66%. It is hoped that this scoring system can be validated in different geographical areas and subsequently be utilized at several places.

## Authors' contributions

All the authors conceptualized the study, participated significantly in the analysis, drafting of the manuscript and writing the final version of the paper. SKM and RM contributed towards the mathematical modeling.
